# Longitudinal links between prenatal maternal depression and anxiety symptoms and infant temperament: The roles of postnatal symptoms and perceived partner support

**DOI:** 10.1002/imhj.70117

**Published:** 2026-09-30

**Authors:** Raphaëlle Giac, Sophie Chaput‐Langlois, Linda Booij, Katherine Séguin, Angélique Brun, Natalie Castellanos‐Ryan, Cathy Vaillancourt, Sarah Lippé, Catherine M. Herba

**Affiliations:** ^1^ Department of Psychology Université du Québec à Montréal Montreal Quebec Canada; ^2^ CHU Sainte‐Justine Azrieli Research Center Montreal Quebec Canada; ^3^ Department of Psychiatry McGill University Montreal Quebec Canada; ^4^ Douglas Research Centre & Eating Disorders Continuum Montreal Quebec Canada; ^5^ Department of Psychoeducation University of Montreal Montreal Quebec Canada; ^6^ INRS ‐ Centre Armand‐Frappier Santé Biotechnologie Laval Quebec Canada; ^7^ Centre de recherche du CIUSSS du Nord‐de‐l’Île‐de‐Montréal Montreal Quebec Canada; ^8^ Department of Psychology University of Montreal Montreal Quebec Canada

**Keywords:** infant temperament, maternal mental health, partner support, perinatal anxiety, perinatal depression

## Abstract

The perinatal period is marked by heightened vulnerability to maternal depression and anxiety symptoms, which have been associated with early child socio‐emotional development, including difficult temperament. This longitudinal study investigated whether prenatal maternal depression and anxiety symptoms predicted infant difficult temperament at 12 months through parallel mediation by postnatal maternal symptoms and perceived partner support. Given emerging evidence on sex differences in the associations between prenatal maternal distress and early socio‐emotional development, exploratory multi‐group analyses by child sex were also conducted. Pregnant individuals were recruited (October 2020–September 2022) for the Resilience and Perinatal Stress during the Pandemic (RESPPA) study in Quebec, Canada. Measures included the EPDS, GAD‐7, ICQ, and perceived partner support assessed via self‐reported online questionnaires. Path analyses (*N = *1422) were conducted using data from the third pregnancy trimester (T1), three months postpartum (T2), and 12 months postpartum (T3). Separate models were estimated for depression and anxiety symptoms. Greater prenatal depression symptoms predicted higher infant difficult temperament scores, with postnatal depression symptoms and perceived partner support fully mediating this association. Similar findings emerged for anxiety. No significant sex differences were observed. Findings highlight postnatal maternal depression and anxiety symptoms and perceived partner support as potential screening and intervention targets to promote infant development.

## 1 INTRODUCTION

The perinatal period, spanning from conception to one year of age (Leiferman et al., 2021), is a time of heightened vulnerability to mental health difficulties for mothers (Cook et al., 2023; Gelaye et al., 2016; McCarthy et al., 2021; Van den Bergh et al., 2020). This period is marked by numerous changes, including hormonal, physical, psychological, financial, and social transitions, which can contribute to the onset or recurrence of mental health challenges (Biaggi et al., 2016; Smith et al., 2011). Maternal mental health problems are considered to be of global public health concern (Howard & Khalifeh, 2020; World Health Organization, 2024). Depression and anxiety symptoms have been found to be the most frequent mental health difficulties reported by women during the perinatal period (Alipour et al., 2012; Mateus et al., 2022). A Canadian study found that one in four women reported symptoms consistent with postpartum depression or postpartum anxiety at five to thirteen months postpartum (Gheorghe et al., 2021). While the perinatal period is already a time of increased vulnerability, the COVID‐19 pandemic likely exacerbated mental health difficulties. In particular, studies reported higher rates of depression and anxiety symptoms among pregnant women following the onset of the pandemic (Abu Sabbah et al., 2022; Caffieri et al., 2024; Tomfohr‐Madsen et al., 2021).

A review including 25 systematic reviews and meta‐analyses conducted during the COVID‐19 pandemic across different countries estimated the pooled prevalence of prenatal and postnatal depression symptoms at 29% and 26%, respectively (Caffieri et al., 2024). Anxiety is also common during pregnancy, with 15% of mothers experiencing an anxiety disorder and 23% reporting elevated symptoms without meeting diagnostic criteria (Dennis et al., 2017). According to findings from a systematic review and meta‐analysis by Nielsen‐Scott et al. (2022), the prevalence of elevated self‐reported anxiety symptoms was 29.2% during the prenatal period and 24.4% during the postnatal period. Additionally, Caffieri et al. (2024) reported a pooled prevalence of 31% for elevated prenatal and postnatal anxiety symptoms in their review. While these results indicate that perinatal depression and anxiety symptoms are highly prevalent, it is important to note that prevalence rates can vary depending on the assessment method and population characteristics (Caffieri et al., 2024). In the context of perinatal mental health, research has disproportionately focused on depression, while anxiety has been comparatively overlooked despite being highly prevalent (Dennis et al., 2017; Howard & Khalifeh, 2020). Studies have shown that elevated depression and anxiety symptoms, even if they do not meet the diagnostic threshold, are a risk factor for less optimal child development (Glover, 2014; Goodman et al., 2011; Herba et al., 2016; Lähdepuro et al., 2023; Letourneau et al., 2019). The present study therefore focuses on symptoms of depression and generalized anxiety, regardless of whether the individual has a diagnosis or not.

### 1.1 Perinatal maternal mental health and child development

The period from conception to the early years of a child's life is a critical period for optimal growth and neurological development throughout the lifetime (Cusick & Georgieff, 2016). Indeed, this is consistent with the Developmental origins of health and disease (DOHaD) hypothesis that suggests that the environment in which individuals develop in utero and during early development can affect their development and later health outcomes (Barker, 1998). The effects on the child may vary depending on the type of in utero exposure and the timing of exposure (Heindel et al., 2015). Studies conducted with both humans and animals have highlighted associations between symptoms of depression and anxiety during pregnancy and fetal as well as child development (Lautarescu et al., 2020; Lupien et al., 2016; Van Den Bergh et al., 2020). Research has documented links with various aspects of child development, particularly the cognitive, motor, and socio‐emotional domains (Buthmann et al., 2022; Duguay et al., 2022; Fiske et al., 2022; Glover, 2014, 2015; Goodman, 2019; Lafortune et al., 2021; O'Connor et al. et al., 2014; Sanger et al., 2015; Schoenmakers et al., 2022). This indicates the relevance of perinatal mental health for child development. However, although the association between prenatal maternal distress and child outcomes is well established, much variability exists as to the extent to which a child might be affected (Herba & Glover, 2021). This variability suggests that contextual or environmental factors can play an important role through exacerbating or mitigating such associations. A better understanding of the pathways linking maternal mental health during pregnancy and early child development is therefore essential for informing more targeted prevention and intervention strategies.

### 1.2 Temperament

A child's temperament is linked to their socio‐emotional adaptation throughout childhood (Buss et al., 2015) and can shape the way they engage with the world, contributing to individual differences in socio‐emotional development (Sanson et al., 2011). Socio‐emotional development encompasses emotional and behavioral problems, as well as difficulties in self‐regulation, processing social information, and understanding emotions (Campbell et al., 2016). Thomas & Chess (1977) introduced a typology of child temperaments: “difficult”, “slow‐to‐warm‐up”, and “easy”. A more difficult temperament is primarily characterized by negative affect (such as irritability and crying), irregular biological functions (e.g., difficulty establishing sleep patterns), withdrawal in the face of novelty, slow adaptability to the environment, and intense reactions/expressiveness. Easy temperament on the other hand, is characterized by positive affect, regular biological functions, approach responses (curiosity or interest in the face of novelty, or the tendency to explore or engage with new stimuli), rapid adaptability, and light or moderate reactions/expressiveness (Thomas & Chess, 1977).

The concept of difficult temperament has been examined in numerous studies (Bhadelia et al., 2024; Hanington et al., 2010; Takegata et al., 2021; Thiel et al., 2021) and has been shown to predict various aspects of children's social and emotional development (Guerin et al., 1997; Patil & Khadi, 2018; Sanson et al., 2011; Wu et al., 2022). A study following 2120 children reported links between a more difficult temperament at 18 months and higher levels of depression and anxiety symptoms at five years of age (Côté et al., 2009). In their longitudinal study, Wu et al. (2022) showed that a difficult temperament at two years of age was positively associated with depression symptoms in adulthood and negatively associated with well‐being in adulthood. It is therefore important to consider early‐life factors, including maternal mental health, that may be linked to a difficult temperament in the infant (Lahey et al., 2008).

**Key findings**

- Greater symptoms of depression during the third trimester of pregnancy were associated with reports of a more difficult infant temperament at 12 months.
- The association between prenatal symptoms of depression and the infant's difficult temperament at 12 months is indirect, mediated through two distinct yet linked pathways: continued postpartum maternal symptoms of depression and lower perceived partner support at three months.
- Similar results were obtained for anxiety symptoms.

**Statement of Relevance**

This study highlights relevant pathways linking prenatal depression and anxiety symptoms and infant difficult temperament, which is a key factor in early emotional development. Postnatal maternal depression and anxiety symptoms as well as perceived partner support were key mediators. By elucidating these pathways, this research contributes to the field of infant and early childhood mental health by informing strategies to mitigate early risk factors and support optimal developmental outcomes.

### 1.3 Prenatal maternal mental health and child temperament

Several studies suggest that prenatal maternal stress, depression, and anxiety may be linked to infant temperament (Gutteling et al., 2005; Korja et al., 2017; Laplante et al., 2016; McGrath et al., 2008; Nomura et al., 2019). Furthermore, prenatal maternal symptoms are often associated with postnatal maternal symptoms, which may partially explain their links to infant development. The neurophysiological processes underlying infant temperament are shaped by both genetic and environmental factors, with the latter including exposure to various forms of stress in utero (Austin et al., 2005; Saudino, 2005; Waxler et al., 2011). Infants exposed to high levels of maternal depression symptoms in utero are reported to have more difficult temperaments at six months of age. For example, they show fewer smiles, laughs, and cuddles, more sadness, more difficulty being comforted, etc. (Nomura et al., 2019). In a study by McGrath and colleagues (2008), reports from non‐depressed mothers about their infant's temperament were significantly different from those of depressed mothers, with the latter evaluating their infants as more difficult at both two and six months of age. Davis and colleagues (2007) also showed that high levels of prenatal maternal anxiety and depression symptoms were significantly associated with the infant's negative reactivity, an aspect of temperament. The association between prenatal depression and infant temperament remained significant after controlling for postnatal anxiety, depression, and stress, while the association between prenatal anxiety and infant temperament became marginal once adjusting for postnatal symptoms (Davis et al., 2007). A study by Sörensen and colleagues (2024) showed that both the timing of maternal symptoms (prenatal vs. postnatal) and the symptom type (anxiety vs. depression) contributed to different temperament trajectories in children. Prenatal anxiety was associated with a high‐rising infant difficult temperament trajectory, whereas prenatal depression/anhedonia symptoms were associated with a stable‐medium trajectory, an association that was attenuated after adjusting for postpartum symptoms. In a study conducted during the pandemic, prenatal maternal distress, measured by anxiety and depression symptoms, was significantly linked to lower socio‐emotional development in infants aged six to 13 weeks (Duguay et al., 2022). However, postnatal distress fully mediated the association between prenatal distress and infant socio‐emotional development, such that the direct effect of prenatal distress was no longer significant (Duguay et al., 2022). This highlights the importance of considering postnatal maternal symptoms when examining the links between prenatal maternal depression and anxiety symptoms and infant outcomes.

In addition to postnatal maternal distress, social support has also been associated with maternal mental health. Social support is a multidimensional concept that can take various forms. Emotional support refers to the direct expression of positive feelings towards someone, reassuring and comforting them. Instrumental support, on the other hand, refers to practical or tangible help (e.g., lending money, assisting with household tasks; House, 1983). Perceived support, the belief that help is available when needed, is the most extensively studied aspect of social support (Yim et al., 2015). Because partner support is a modifiable protective factor, it constitutes a central element in preventive strategies for perinatal depression and anxiety (Dennis & Ross, 2006; Highet et al., 2011; Hopkins & Campbell, 2008). Partner support is often identified as one of the main protective factors against maternal psychological distress (Lebel et al., 2020; Stapleton et al., 2012). Several studies have reported cross‐sectional associations between social support and maternal mental health, particularly depression symptoms (Cheng et al., 2016; Dibaba et al., 2013; Lau et al., 2014; Li et al., 2021). In a systematic review, Antoniou and colleagues (2021) concluded that there is a strong link between low partner support, both emotional and instrumental, and symptoms of anxiety and depression in women during the perinatal period. Stapleton et al. (2012) examined these associations longitudinally. They found that mothers who perceived greater support from their partner in mid‐pregnancy experienced less emotional distress (anxiety and depression symptoms) after childbirth (after controlling for their distress at the beginning of pregnancy). Greater partner support also predicted lower infant novelty reactivity, an aspect of the child's temperament (Stapleton et al., 2012).

However, most studies examining partner support and perinatal mental health have focused on how partner support predicts later maternal symptoms or is associated with them at the same time (Antoniou et al., 2021; Stapleton et al., 2012). Yet, the reverse association is also plausible. Prenatal maternal depression symptoms could themselves lead to a deterioration in perceived partner support over time. Senturk et al. (2017) directly investigated this possibility in a longitudinal study assessing women during the third trimester and again postnatally at two, 12 and 18 months. They found that perceived emotional support from the partner declined more strongly in women with prenatal (third trimester) depressive symptoms. This may be due to maternal depression symptoms negatively affecting the couple's relationship, or to the fact that depressed individuals may be less likely to recognize or perceive the support that is available to them. Despite the plausibility of such effects, very few studies have explored this direction of influence.

In addition to its direct associations with maternal symptoms, perceived partner support may also play a mediating role in the transmission of risk from prenatal maternal distress to infant outcomes. Several possible mechanisms may be operating to explain the importance of the perceived partner's support. Within the framework of Goodman & Gotlib's (1999) model, the intergenerational transmission of depressive risk is based on multiple mechanisms including a stressful environment. In this context, perceived partner support may contribute to intermediate factors, such as less maternal emotional distress, better maternal emotion regulation or more sensitive and positive mother‐child interactions. Through these pathways, perceived partner support could be indirectly linked with infant temperament via environment and parental behavior mechanisms (Martin & Brock, 2023; Zhang et al., 2019). Consistent with this broader developmental perspective, Schuijers et al. (2024) reported that greater maternal perinatal social support was associated with fewer social‐emotional problems and greater social‐emotional competencies in one‐year‐old offspring, providing longitudinal evidence linking maternal support to early socio‐emotional development. Further, De Waal et al. (2023) found an indirect effect of postnatal (at eight months) perceived partner support on child social‐emotional development (at two years old), but only indirectly through mother–infant bonding at eight months postpartum. This mediating role of perceived partner support integrates a relational dimension into Goodman and Gotlib's model, highlighting the importance of the social context in the transmission of depressive risk. More recently, Curci and colleagues (2025) extended this literature by examining perceived partner support as a moderator of the association between postpartum depressive symptoms and mother–infant dyadic reciprocity. Their findings suggest that partner support may buffer the negative impact of maternal emotional distress on early mother–infant relational processes, highlighting its role not only as a predictor but also as a contextual protective factor within postpartum adjustment.

Although studies have suggested a link between perceived partner support and symptoms of emotional distress during pregnancy (Antoniou et al., 2021; Cheng et al., 2016) and postpartum (Milgrom et al., 2008; Okatvia & Kanathasan, 2025; Stapleton et al., 2012), to our knowledge, no study has examined the mediating role of perceived partner support in the relationship between prenatal maternal depression and anxiety symptoms and reported infant difficult temperament, while simultaneously considering the role of postnatal maternal symptoms. Yet, both perceived partner support and postnatal maternal distress are interrelated and theoretically relevant variables, suggesting that they should be examined together in models seeking to understand how early maternal experiences can be linked to infant temperament.

Finally, studies have highlighted the importance of considering the child's sex when examining the associations between prenatal maternal distress and child health outcomes (Bale, 2011; Clifton, 2010; Glover & Hill, 2012; Sutherland & Brunwasser, 2018). Emerging evidence suggests that prenatal maternal distress may be differentially associated with socio‐emotional development in males and females, potentially due to sex‐specific fetal adaptation processes, differences in placental functioning, and stress‐response systems (Bale, 2011; Braithwaite et al., 2020; Clifton, 2010; Galbally et al., 2022). Some studies have reported stronger associations between prenatal maternal distress and later emotional or internalizing difficulties among female offspring (Braithwaite et al., 2020; Galbally et al., 2022). In contrast, other studies have reported relatively limited sex differences in the longitudinal pathways linking maternal anxiety, child temperament, and later socio‐emotional functioning during early childhood (Behrendt et al., 2020). However, the findings to date tend to be mixed, likely due to variations in the timing of exposure, the specific outcome assessed, and the age at assessment (Glover & Hill, 2012; Sutherland & Brunwasser, 2018).

### 1.4 Current study

The goal of this study was to examine the longitudinal associations between maternal depression and anxiety symptoms in late pregnancy and the infant's difficult temperament at 12 months. The mediating roles of maternal symptoms and perceived partner support at three months postpartum were also considered. Finally, we tested whether the infant's sex moderated effects. Models were run separately for depression symptoms and anxiety symptoms in order to more carefully understand the contribution of each symptom type.

The primary hypothesis was that higher maternal depression and anxiety symptoms during the third trimester of pregnancy would be associated with a more difficult infant temperament at 12 months. The second hypothesis was that this link would be mediated through maternal depression and anxiety symptoms as well as perceived partner support at three months postpartum. Specifically, we expected that higher prenatal depression and anxiety symptoms would be associated with higher depression and anxiety symptoms and lower perceived partner support at three months postpartum, which in turn would be associated with a more difficult infant temperament at 12 months. The moderating role of the infant's sex was exploratory. Since all adult participants in this study identified as female and were mothers, we used the terms “women” and “mothers” throughout this paper to align with the terminology commonly used in the literature.

## 2 METHODS

### 2.1 Study design and participants

Our objectives were addressed within the context of the longitudinal Resilience and Perinatal Stress during the Pandemic (RESPPA) study, whereby women were recruited during pregnancy or within three months of giving birth and followed for up to two years postnatally across different regions of the province of Quebec, Canada (Brun et al., 2025; Séguin et al., 2026). The cohort focused on several health areas, such as mental health, nutrition, and physical exercise. Data were captured via convenience sampling and a total of 1669 participants were recruited between October 2020 to September 2022 via advertisements on social media (Facebook and Instagram) or at one of seven study sites: CHU Sainte‐Justine; CHU de Québec; CIUSSS de l'Estrie; CIUSSS du Nord‐de‐l'île‐de‐Montréal; CIUSSS de la Mauricie‐et‐du‐Centre‐du‐Québec; CISSS de l'Abitibi‐Témiscamingue; CISSS du Bas‐Saint‐Laurent. The inclusion criteria for this study were (i) being pregnant or less than three months postpartum, (ii) being at least 18 years old, (iii) residing in the province of Quebec and (iv) being able to complete online questionnaires in French or English. Participants were excluded from current analyses if they gave birth to twins (*n *= 9), experienced pregnancy loss or loss of the infant shortly after birth (*n *= 60), or had an infant born under 28 weeks (*n *= 1), a gestational age classified as extremely preterm by the World Health Organization (WHO, 2023). The present study used data from at least one of the following time points: the third trimester of pregnancy (T1), three months (T2), and 12 months (T3) after delivery, with the final sample for analyses consisting of 1422 women. Participants provided informed consent, and the study was approved by the ethics committee of the central site CHU Sainte‐Justine and participating hospital sites. While the study was conducted within the context of the pandemic, we were not testing pandemic effects per se.

### 2.2 Measures

Self‐reported online questionnaires were administered via Limesurvey and completed in either French or English, depending on the participant's preference.

#### 2.2.1 Maternal depression

Depression symptoms were measured via the *Edinburgh Postnatal Depression Scale* (EPDS; Cox et al., 1987). The EPDS is the most widely used measure to assess depression symptoms during the perinatal period (Gollan et al., 2017; Moyer et al., 2024). The scale contains ten items and assesses the severity of depression symptoms over the last seven days (e.g., “In the past seven days, I have been so unhappy that I have been crying”). Each item is scored on a four‐point Likert scale, ranging from 0 to 3, with a higher score indicating greater symptoms of depression. To account for possible missing values on individual items, a mean score was calculated for participants who completed at least seven of the ten items, and this score was multiplied by the total number of questions, yielding a total score ranging from 0 to 30. In our sample, the EPDS had good internal consistency (Cronbach's α at both T1 and T2 = .87). In line with established clinical cut‐off scores, an EPDS score ≥ 11 was used to identify participants with clinically significant depressive symptoms (Levis et al., 2020).

#### 2.2.2 Maternal anxiety

The *Generalized Anxiety Disorder‐7* questionnaire (GAD‐7; Spitzer et al., 2006) includes seven items and was administered to measure the severity of maternal anxiety symptoms over the last two weeks. This measure has been well validated in several populations worldwide, including women in the perinatal period (Simpson et al., 2014). Participants were asked to rate how often they had been bothered by each of the following seven items (e.g., “Feeling nervous, anxious, or on edge”) over the past two weeks. Each item is evaluated using a four‐point Likert scale ranging from 0 to 3 (0 = never to 3 = almost every day). The total score thus ranged from 0 to 21. To account for possible missing values on individual items, a mean score was calculated for participants who completed at least five of the seven items, and this score was multiplied by the total number of questions. In our sample, the GAD‐7 had excellent internal consistency (Cronbach's α at T1 = .90, Cronbach's α at T2 = .91). A GAD‐7 score ≥ 10 was used to identify participants with clinically significant anxiety symptoms (Spitzer et al., 2006).

#### 2.2.3 Infant temperament

Mothers reported on their infant's difficult temperament at 12 months (T3) using the *Infant Characteristics Questionnaire* (ICQ; Bates et al., 1979). The ICQ is a scale composed of 32 items grouped under four components of child temperament: 1) Fussy‐Difficult (e.g., cries often and is difficult to console) 2) Unadaptable (e.g., struggles to adjust to new places or people) 3) Dull (e.g., shows minimal reactions to stimuli, seems less interested in their environment) 4) Unpredictable (e.g., difficulty in predicting baby's needs, such as hunger) (Bates et al., 1979). To assess the infant's difficult temperament, we used the Fussy‐Difficult component, which consists of nine items (e.g., “How easy is it for you to calm or soothe your child when he/she is upset”; Bates et al., 1979). Each item is rated on a seven‐point Likert scale, where a higher score indicates a more difficult temperament. To account for possible missing values on individual items, a mean score was calculated for participants who completed at least seven of the nine items, and this score was multiplied by the total number of questions. In our sample, the component Fussy‐Difficult of the ICQ demonstrated good internal consistency (Cronbach's α at T3 = .85).

#### 2.2.4 Perceived partner support

Perceived partner support was measured using five items developed by Saysset, Boivin and Piché and used in the Quebec Longitudinal Study of Child Development (QLSCD; Institut de la statistique du Québec, 2022, p. 47; Saysset et al., 1998). The scale assesses two dimensions of partner support in various situations: instrumental and emotional. Two items focus on instrumental support (e.g., “To what extent do you feel supported by your current partner in the household chores?”), two on emotional support (e.g., “To what extent do you feel supported by your current partner when you are overwhelmed?”) and one on general support. Participants responded using a 10‐point Likert scale ranging from 1 to 10 (1 = Not at all to 10 = Very much so). Perceived partner support was assessed at three months postpartum (T2). A mean score was calculated for the participants who completed at least four of the five items. In our sample, this instrument had excellent internal consistency (Cronbach's α at T2 = .90).

### 2.3 Analysis plan

Descriptive statistics and correlations were conducted with IBM SPSS Statistics 29 (IBM Corp., 2023). Main analyses were then performed using Mplus 8.11 (Muthén & Muthén, 2024) with data collected from the third trimester of pregnancy (T1), at three months postpartum (T2) and 12 months postpartum (T3). First, a path analysis model was conducted to examine the links between prenatal depression symptoms and the infant's difficult temperament at 12 months postnatal. Then, a parallel mediation model with two mediators was tested to determine whether the effect of prenatal maternal depression on the infant's difficult temperament at 12 months postnatal was explained, fully or in part, by two distinct but simultaneous mediators assessed at 3 months postnatally: maternal depression symptoms and perceived partner support. This approach allows for the evaluation of the independent role of each mediator while accounting for their coexistence within the model. Finally, a multiple group analysis was performed to test if the mediation model differed between boys and girls. A chi‐square difference test was used to determine if constraining the links led to a worse fit, which would mean that differences between sexes existed. A second similar model was tested, this time focusing on anxiety symptoms. Given the high correlation between depression and anxiety symptoms at each time point (*r* = .78–.77, *p* < .001), they were examined in separate models to capture their potentially distinct and complementary associations with the outcome. In all models tested, the following covariates were included: the mother's age at the birth of the child, education level, use of selective serotonin reuptake inhibitors (SSRIs) and/or benzodiazepines during the third trimester of pregnancy, the baby's gestational age at birth, if the pregnancy was planned, if it was their first pregnancy and if the baby has been breastfed up until three months. These were selected based on their theoretical relevance and their strength of correlation with variables of interest. For gestational age of the baby at birth, univariate outliers (*n *= 14) were winsorized at 3.29 standard deviations from the mean (Wilcox, 2003). Data for these covariates were collected through self‐reported online questionnaires. Pearson's correlations and descriptive statistics among variables of interest are presented in Table 2. Sociodemographic and pregnancy status variables were weakly to moderately associated with maternal prenatal depression and anxiety symptoms and infant temperament (see Table 2). Partner support showed the strongest associations, with correlations varying from *r *= −.19 to *r *= −.39. Infant sex was not associated with any of the main study variables.

Model fit was assessed using several indices, including the chi‐square test, the Comparative Fit Index (CFI; value ≥ .95 represents an excellent fit), the Root Mean Square Error of Approximation (RMSEA; value < .05 represents an excellent fit), and the Standardized Root Mean Square Residual (SRMR; value < .08 represents a good fit; Schermelleh‐Engel et al., 2003). All reported results are based on the standardized model results (i.e., standardized estimates are reported). For the parallel mediation model, the bootstrapping method was used to estimate the indirect effect and the index of moderated mediation, generating 95% bias‐corrected confidence intervals (CI) using 5000 samples to determine whether the effect was significant. Significance was achieved when the 95% CI did not include zero. A power calculation was conducted using Monte Carlo simulations in Mplus version 8.11 (Muthén & Muthén, 2002). With a sample size of 1422 participants, it will be possible to detect small indirect effects (β = .10) with 80% power and a Type I error rate of 5%.

### 2.4 Missing data

We examined whether missing data were associated with any of the study variables. Of the participants, 62% had missing values on at least one study variable, whereas 38% had complete data. Participants with missing data reported slightly higher maternal depression symptoms at T1 compared to those without missing data: *t*(1122.991) = 2.87, *p* = .004, M_missing_ = .81, M_no missing_ = .72. The difference in maternal depression symptoms at T1 represented a trivial effect size (*d* = .17), and no significant differences were found for maternal depression at T2.  Those with missing data also showed lower maternal age and education: *t_age_* (1411) = −3.06, *p* = .002, M_missing_ = 30.82, M_no missing_ = 31.54; *t_education_* (1218.310) = −6.06, *p* < .001, M_missing_ = 3.99, M_no missing_ = 4.52. No other significant differences were found. At T1, 1134 participants had available data on at least one measure of depression or anxiety symptoms. At T2, 1070 participants had available data on at least one measure of depression or anxiety symptoms or partner support. At T3, 812 participants had available data on the difficult temperament measure. To account for missing data, Full Information Maximum Likelihood estimation (FIML) was used, allowing the inclusion of all available data from participants.

## 3 RESULTS

### 3.1 Participant characteristics

Our sample included 1422 participants. Descriptive information can be found in Table 1. Mean age of mothers at the birth of their child was 31 years (SD = 4.31; range 19–44 years). Most participants were married or in a common‐law relationship (93.4%), identified as White (95.5%), had a bachelor's (30.7%) or post‐graduate degree (26.3%), and reported an annual household income of $100,000 CAD or more (55.9%). Just over half were experiencing their first pregnancy (53.6%), and most pregnancies were planned (85.2%). The vast majority (89.8%) did not use selective serotonin reuptake inhibitors (SSRIs) and/or benzodiazepines during the third trimester of pregnancy. Infants were relatively evenly distributed by sex (53.4% boys; 46.6% girls), and the majority were breastfed up until three months postpartum (89.2%). Mean gestational age of the baby at birth was 39 weeks (SD = 1.39; range 34–42 weeks). Pearson's correlations and descriptive statistics among variables of interest are presented in Table 2. For depression symptoms, 27.2% of participants scored above the threshold for clinically significant symptoms on the EPDS at T1, and 23.3% at T2. For anxiety symptoms, 14% of participants scored above the clinical cut‐off on the GAD‐7 at T1, and 13.6% at T2.

**TABLE 1:** Demographic characteristics of the study participants (*N *= 1422).

Characteristic	n (%)
*Mother's age at birth of baby (years)*	
From 18 to 29	561 (39.5%)
From 30 to 39	837 (59.0%)
40 and above	21 (1.5%)
Missing	3
*Marital status*	
Married/Common‐law	1323 (93.4%)
Single	82 (5.8%)
Separated or divorced	12 (.8%)
Missing	5
*First pregnancy*	
Yes	663 (53.6%)
No	573 (46.4%)
Missing	186
*Planned pregnancy*	
Yes	1052 (85.2%)
No	183 (14.8%)
Missing	187
*Highest level of education*	
High school diploma or less	93 (6.9%)
Trade school diploma or CÉGEP^1^ diploma	411 (30.3%)
University diploma below a bachelor	79 (5.8%)
Bachelor's degree	416 (30.7%)
Postgraduate degree (Master, Ph.D.)	356 (26.3%)
Missing	67
*Annual household income (before tax in Canadian dollars)*	
Less than 10,000$	8 (.6%)
10,000 – 29,999$	47 (3.5%)
30,000 – 49,999$	95 (7.1%)
50,000 – 69,999$	123 (9.2%)
70,000 – 99,999$	319 (23.8%)
100,000$ or more	750 (55.9%)
Missing	80
*Ethnicity of the mother*	
White	1304 (95.5%)
Black	21 (1.5%)
Latin American	20 (1.5%)
Asian	13 (1%)
Arab	4 (.3%)
Indigenous	4 (.3%)
Missing	56
*Use of SSRIs* ^2^ *and/or benzodiazepines during the 3rd trimester of pregnancy*	
No	994 (89.8%)
Yes	113 (10.2%)
Missing	315
*Baby was breastfed up until three months postpartum*	
Yes	847 (89.2%)
No	103 (10.8%)
Missing	472
*Gestational age of the baby at birth*	
34 to 36 weeks	53 (4.6%)
37 to 39 weeks	679 (57.9%)
40 to 42 weeks	442 (37.7%)
Missing	248
*Infant sex*	
Boys	627 (53.4%)
Girls	548 (46.6%)
Missing	247
*Depression score (EPDS) T1*	
Under clinical cut‐off (< 11)	819 (72.8%)
Over clinical cut‐off (≥ 11)	306 (27.2%)
*Depression score (EPDS) T2*	
Under clinical cut‐off (< 11)	817 (76.7%)
Over clinical cut‐off (≥ 11)	248 (23.3%)
*Anxiety score (GAD‐7) T1*	
Under clinical cut‐off (< 10)	974 (86%)
Over clinical cut‐off (≥ 10)	160 (14%)
*Anxiety score (GAD‐7) T2*	
Under clinical cut‐off (< 10)	924 (86.4%)
Over clinical cut‐off (≥ 10)	146 (13.6%)

^1^ CÉGEP refers to a post‐secondary education system unique to Quebec, designed as an intermediary step between high school and either university or technical training. It offers two main types of programs: two‐year pre‐university programs or three‐year technical programs.

^2^ Selective Serotonin Reuptake Inhibitors.

**TABLE 2:** Descriptive statistics and correlations among study variables.

	1	2	3	4	5	6	7	8	9	10	11	12	13	14
Depression^1^														
1. T1														
2. T2	.56^***^													
Anxiety^2^														
3. T1	.78^***^	.49^***^												
4. T2	.48^***^	.77^***^	.54^***^											
5. Difficult temperament^3^ (T3)	.18^***^	.19^***^	.13^***^	.16^***^										
6. Infants’ sex	−.01	−.02	−.03	−.01	.03									
7. Partner support (T2)	−.27^***^	−.39^***^	−.21^***^	−.31^***^	−.19^***^	.05								
8. Mother's age	−.07^*^	−.07^*^	−.12^***^	−.06	−.01	.03	.01							
9. Education level	−.21^***^	−.21^***^	−.19^***^	−.18^***^	.03	−.02	.11^***^	.28^***^						
10. Baby's gestational age	−.08^*^	−.09^**^	−.07^*^	−.09^**^	−.04	.01	.02	−.03	.10^***^					
11. SSRI/Benzo use	.10^***^	.10^**^	.13^***^	.13^***^	.08^*^	−.02	.01	.04	−.09^**^	−.15^***^				
12. Planned pregnancy	.14^***^	.15^***^	.15^***^	.13^***^	−.02	−.01	−.16^***^	−.12^***^	−.17^***^	−.01	.01			
13. First pregnancy	.10^***^	.06	.07^*^	.05	.09^*^	.04	−.08^*^	.30^***^	−.03	−.01	−.01	−.03		
14. Breastfeeding	−.00	−.06	.05	−.00	.12^**^	−.01	−.02	.04	.20^***^	.08^*^	−.02	−.09^**^	.05	
*N*	1125	1065	1134	1070	812	1175	956	1419	1355	1174	1107	1235	1236	950
*Mean*	7.65	7.06	7.35	6.92	28.90	–	7.70	31.11	–	39.02	–	–	–	–
*SD*	5.37	5.15	6.41	6.42	8.31	–	1.94	4.31	–	1.39	–	–	–	–

*Note*.

^1^ A mean score of depression was computed with the *Edinburgh Postnatal Depression Scale*.

^2^ A mean score of anxiety was computed with the *Generalized Anxiety Disorder*‐*7* questionnaire.

^3^ A mean score of the nine items related to the difficult temperament dimension from the *Infant Characteristics Questionnaire* were computed. Planned pregnancy and first pregnancy were coded as 1 = yes and 2 = no. At 3 months postnatal, mothers reported on their breastfeeding (including pumped milk fed to baby in a bottle); yes = 1; never or breastfed 1 time = 0. T1 = Time 1 (third pregnancy trimester); T2 = Time 2 (3 months postnatal); T3 = Time 3 (12 months postnatal).

*** *p* < .001

** *p* < .01

* *p* < .05

### 3.2 Main analyses

#### 3.2.1 Depression model

All models showed an overall excellent fit to the data (see Table 3). As shown in Figure 1, prenatal depression symptoms (T1) were significantly and positively associated with mother‐reported infant difficult temperament at 12 months (T3) (c: β = .165, 95% C.I. [.089, .242], *p* < .001). Prenatal depression symptoms (T1) were also significantly and positively associated with postnatal depression symptoms (T2) (β = .541, 95% C.I. [.481, .597], p < .001), reflecting a large effect size. In turn, postnatal depression symptoms (T2) were significantly and positively associated with difficult infant temperament (T3) (β = .145, 95% C.I. [.039, .243], p = .005), reflecting a small effect size. Greater prenatal depression symptoms at T1 were also significantly associated with less perceived partner support at T2 (β = −.253, 95% C.I. [−.327, −.175], p < .001), reflecting a small‐to‐moderate effect size. In turn, lower perceived partner support at T2 was significantly associated with higher scores on the infant's difficult temperament at T3 (β = −.129, 95% C.I. [−.221, −.038], p = .006), reflecting a small effect size. Once both mediators were included in the model, the direct association from prenatal depression to the infant's difficult temperament became non‐significant (c’: β = .053, 95% C.I. [−.038, .147], p = .266). A significant negative correlation was observed between the two mediators (at T2), such that higher postnatal depression symptoms were linked with lower perceived partner support (r = −.283, p < .001). Thus, results indicated that greater postnatal depression symptoms and lower perceived partner support fully mediated the link between prenatal depression symptoms and the infant's difficult temperament. Overall, 67.7% of the total effect between prenatal depression and the infant's difficult temperament was explained by indirect effects, with 47.6% mediated through postnatal depression symptoms and 20.1% through perceived partner support. The betas used to calculate these percentages can be found in Table 4. Multigroup analyses showed that the model did not significantly differ between boys and girls (free model: χ^2 ^= 34.936, df = 42; constrained model: χ^2 ^= 61.154, df = 60; Δχ^2^ = 26.218, Δdf = 18, p > .05).

**TABLE 3:** Model fit of the direct and mediation models for depression and anxiety.

	x^2^ (df)	*p*	RMSEA	CFI	TLI	SRMR
Direct model—path analysis						
Depression (model 1)	10.90 (12)	.5371	.000	1.000	1.000	.015
Anxiety (model 2)	12.98 (12)	.3709	.008	.959	.973	.016
Parallel mediation model						
Depression (model 1)	22.48 (21)	.3725	.007	.997	.997	.017
Anxiety (model 2)	22.70 (21)	.3604	.008	.996	.995	.018

*Note*. RMSEA = Root Mean Square Error of Approximation; CFI = Comparative Fit Index; TLI = Tucker‐Lewis Index; SRMR = Standardized Root Mean Square Residual.

**FIGURE 1:**
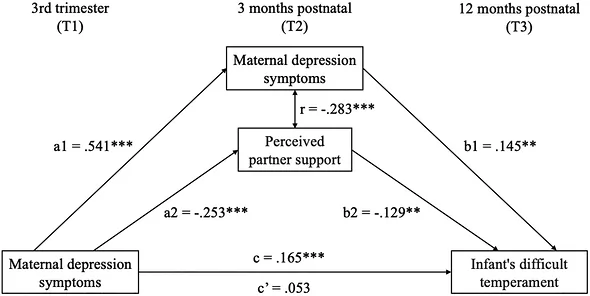
Parallel mediation model for maternal depression symptoms (model 1) *** p < .001 ** p < .01 * p < .05 . Note: N = 1422; a1, a2, b1, b2, c and c’ represent unstandardized path coefficients. Path c represents the total effect of maternal depression symptoms (T1) on the infant's difficult temperament (T3), whereas path c’ reflects the direct effect after accounting for the mediators. Path r represents the correlation between the two mediators measured at T2. Covariates include the age of the mother when the child was born and her level of education, the use of SSRIs and/or benzodiazepines during the 3rd trimester of pregnancy, the baby's gestational age at birth, if the pregnancy was planned, if it was their first pregnancy and if the baby was breastfed up until three months.

**TABLE 4:** Parallel mediation analysis

	Depression		Anxiety	
Path	β	Bias corrected 95% C.I.	β	Bias corrected 95% C.I.
Total effect	.164	.089–.240	.113	.035–.193
Direct effect	.053	‐.038–.147	.022	‐.070–.114
Total indirect effect	.111	.056–.166	.091	.039–.147
Indirect effects				
Postnatal depression	.078	.022–.135	.061	.009–.118
Perceived partner support	.033	.009–.062	.030	.012–.057

#### 3.2.2 Anxiety model

As shown in Figure 2, prenatal anxiety symptoms (T1) were significantly and positively associated with the infant's difficult temperament at 12 months (T3) (c: β = .113, 95% C.I. [.035, .190], p < .01). Prenatal anxiety symptoms (T1) were also significantly and positively associated with postnatal anxiety symptoms (T2) (β = .527, 95% C.I. [.465, .589], p < .001), reflecting a large effect size. In turn, postnatal anxiety (T2) was significantly and positively associated with the infant's difficult temperament (T3) (β = .116, 95% C.I. [.015, .219], p = .026), reflecting a small effect size. Prenatal anxiety was also significantly associated with lower perceived partner support (T2) (β = −.192, 95% C.I. [−.268, −.118], p < .001), reflecting a small effect size. In turn, perceived partner support was significantly and negatively associated with the infant's difficult temperament (β = −.156, 95% C.I. [−.245, −.062], p < .001), reflecting a small effect size. Similar to the depression model, once the two mediators were included in the model, the direct association from prenatal anxiety to the infant's difficult temperament became non‐significant (c’: β = .022, 95% C.I. [−.070, .114], p = .642). There was a significant negative correlation between the mediators (i.e. postnatal anxiety and perceived partner support; r = −.221, p < .001). Greater postnatal anxiety symptoms and lower perceived partner support fully mediated the link between prenatal anxiety symptoms and the infant's difficult temperament. Overall, 80.5% of the total effect between prenatal anxiety and the infant's difficult temperament was explained by indirect effects, with 54% mediated through postnatal anxiety symptoms and 26.5% through perceived partner support. The betas used to calculate these percentages can be found in Table 4. Multigroup analyses showed that the model did not significantly differ between boys and girls (free model: χ^2^ = 34.862, df = 42; constrained model: χ^2^ = 44.794, df = 60; Δχ^2^ = 9.932, Δdf = 18, p > .05).

**FIGURE 2:**
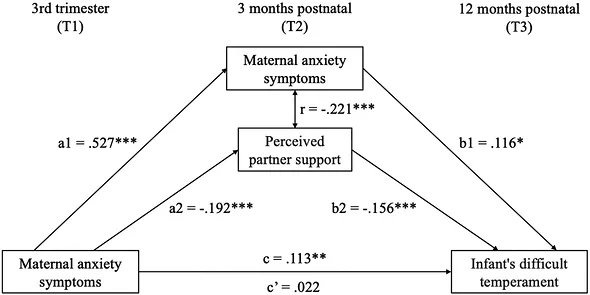
Parallel mediation model for maternal anxiety symptoms (model 2) *** p < .001 ** p < .01 * p < .05 . Note: N = 1422; a1, a2, b1, b2, c and c’ represent unstandardized path coefficients. Path c represents the total effect of maternal anxiety symptoms (T1) on the infant's difficult temperament (T3), whereas path c’ reflects the direct effect after accounting for the mediators. Path r represents the correlation between the two mediators measured at T2. Covariates include the age of the mother when the child was born and her level of education, the use of SSRIs and/or benzodiazepines during the 3rd trimester of pregnancy, the baby's gestational age at birth, if the pregnancy was planned, if it was their first pregnancy and if the baby was breastfed up until three months.

## 4 DISCUSSION

This study sought to investigate the longitudinal associations between prenatal maternal depression and anxiety symptoms and reported infant difficult temperament at 12 months, while examining pathways through potential mediators such as postnatal symptoms (depression or anxiety) and perceived partner support at three months postpartum. Infant sex was also tested as a moderator.

In line with study hypotheses, findings indicated that maternal depression symptoms in late pregnancy were associated with mother‐reported infant difficult temperament at 12 months. Similar results were obtained in the model examining anxiety symptoms. These findings are consistent with those of Austin et al. (2005), reporting associations between prenatal (third trimester) maternal depression symptoms and infant difficult temperament. In the same study, associations were also found between prenatal maternal trait anxiety and infant difficult temperament. Whereas our study assessed infant difficult temperament at 12 months using the Infant Characteristics Questionnaire (ICQ), their study used the Short Infant Temperament Questionnaire at four or six months (Sanson et al., 1987). As in our study, depression symptoms were measured during the third trimester with the EPDS. Whereas we assessed generalized anxiety symptoms using the GAD‐7, they measured trait anxiety symptoms using the trait component of the State‐Trait Anxiety Inventory (STAI) (Spielberger, 1983). McMahon et al. (2013) also reported associations between trait anxiety during the third trimester of pregnancy and infant difficult temperament at four months. Trait anxiety and infant difficult temperament were measured using the STAI and the Short Infant Temperament Questionnaire, respectively. McGrath et al. (2008) categorized participants into depressed and non‐depressed groups on their Center for Epidemiologic Studies Depression Scale scores during the third trimester of pregnancy and found that mothers with depression both before and after giving birth viewed their infants as more difficult at two and six months old than mothers without prenatal depression. Contrary to our study, all the studies discussed in the present paragraph tested direct links between psychological distress in pregnancy and infant difficult temperament. Interestingly, however, McMahon et al. (2013) found that maternal trait anxiety, and not state anxiety or pregnancy‐related anxiety, measured during pregnancy was related to infant difficult temperament. This finding suggested that women with more stable tendencies to feel anxious were more likely to report that their infant had a difficult temperament. While their study did not formally test mediation, it supports the idea that not all forms of psychological distress in pregnancy are similarly linked to infant temperament. From a theoretical perspective, results from the present study are consistent with developmental programming models, including the Developmental Origins of Health and Disease (DOHaD) framework. According to this perspective, exposure to maternal psychological distress during sensitive periods of prenatal development may influence the maturation of fetal neurobiological systems involved in emotional reactivity and stress regulation. Infant difficult temperament may therefore reflect early‐emerging differences in emotional reactivity and self‐regulatory capacities that are shaped by prenatal environmental influences.

Importantly, the present findings suggest that these prenatal influences do not operate in isolation but rather unfold through postnatal psychological and relational processes. This is in line with our results, indicating the importance of considering relevant postnatal mediating factors as pathways linking prenatal distress to infant temperament. This idea is further elaborated in the following section, which highlights the role of postnatal factors in explaining this association.

Greater maternal depression and anxiety symptoms and lower perceived partner support at three months mediated links between depression and anxiety symptoms during the third pregnancy trimester and the infant's difficult temperament at 12 months. While both postnatal factors mediated associations, results appeared stronger for the pathway through postnatal symptoms rather than perceived partner support. Our results are in line with another study conducted during the pandemic. Specifically, in Duguay et al.’s (2022) study, postnatal depression and anxiety symptoms fully mediated the association between prenatal distress and infants' socio‐emotional development between six and 13 weeks postpartum, such that the direct effect of prenatal distress was no longer significant. However, their study assessed infant socio‐emotional difficulties using the Ages and Stages Questionnaire (ASQ; Squires et al., 2015) whereas we focused more specifically on difficult temperament. Further, their measurements were taken between six and 13 weeks postpartum, whereas we evaluated temperament at 12 months postpartum. Importantly, we also considered two relevant mediators in parallel at three months postpartum, taking both into account within the same model. A key contribution of our study to the literature is its clear distinction between prenatal and postnatal maternal distress in relation to the child's social‐emotional development. These results are also consistent with Vismara et al.’s (2021) study which showed that maternal trait anxiety and depression measured at three months postpartum were the most significant predictors of mother‐reported infant negative affectivity over prenatal trait anxiety. However, pre‐ and postnatal mental health variables were moderately to highly correlated, highlighting the relevance of considering postnatal maternal symptoms as a pathway linking prenatal depression or anxiety to infant temperament.

As for the mediating role of perceived partner support, it may be hypothesized that when mothers perceive greater partner support, it reflects a partner who is more involved in domestic and parenting responsibilities, which may in turn be associated with more positive developmental outcomes. Within Goodman & Gotlib's (1999) model of intergenerational transmission of depression, environmental stressors and interpersonal processes are conceptualized as key mechanisms linking maternal vulnerability to child outcomes. Perceived partner support can be understood as an important component of the broader caregiving environment. The present findings suggest that partner support may represent an important relational resource within this environment. Although our study did not examine whether partner support moderates the association between prenatal distress and infant temperament, the observed indirect association through perceived partner support is consistent with the possibility that partner support may influence infant development through its effects on maternal emotional functioning and caregiving processes. In other words, although these mechanisms were not directly examined in the present study, perceived partner support may influence infant development indirectly through processes such as maternal affect regulation, perceived caregiving burden, and the quality of dyadic interactions. Although these mechanisms were not directly examined in our study, our findings are consistent with the possibility that perceived partner support contributes to infant socio‐emotional development through its influence on maternal psychological functioning and caregiving processes. The present results are consistent with those of De Waal et al. (2023), who found an indirect effect of perceived partner support at eight months postpartum on child socio‐emotional development at two years of age, mediated through mother–infant bonding. Although mother–infant bonding was not examined in the present study, these findings are consistent with the possibility that relational processes may represent important pathways through which partner support contributes to child socio‐emotional development. Such findings point to possible explanatory pathways that warrant further investigation.

Taken together, the present results are consistent with a developmental cascade framework in which prenatal maternal distress may initiate a sequence of vulnerabilities that unfold through postnatal psychological and relational processes, potentially contributing to differences in infant socio‐emotional development. Rather than reflecting a single direct effect of prenatal symptoms on infant outcomes, the findings are consistent with the notion that the association unfolds through interconnected psychological and relational processes occurring across the perinatal period.

There was no evidence that models differed between boys or girls, when considering models examining either maternal depression or anxiety symptoms. Prior research on this topic has yielded inconsistent findings (Bale, 2011; Clifton, 2010; Glover & Hill, 2012; Sutherland & Brunwasser, 2018), suggesting that such differences may depend on the specific outcome or developmental age assessed. This underscores the complexity of sex‐dependent effects. Indeed, factors such as the nature and timing of maternal distress exposure, the specific outcomes measured, the developmental stage at assessment, and even species differences may all contribute to variations in sex‐related findings (Glover & Hill, 2012).

### 4.1 Limitations and strengths

The present study had various strengths such as the large sample and longitudinal design spanning late pregnancy to the first postpartum year. By collecting data at three key time points, we were able to assess the temporal relationships between variables and better understand the mechanisms underlying the relation between perinatal maternal depression and anxiety symptoms and infant difficult temperament. We also considered the role of two relevant mediating variables, that of postnatal symptoms (depression or anxiety depending on the model) and perceived partner support, within the same model so that we could account for the correlation between the two mediators. Another strength of this study lies in considering not only maternal depression symptoms but also anxiety symptoms, which are just as prevalent in the perinatal period but less studied. This allowed for a more comprehensive exploration of these two forms of distress.

However, findings from the current study should be interpreted considering the following limitations. First, it is important to highlight the subjectivity of the infant temperament measure, which is self‐reported by the mother and thus reflects her perception of her infant. Nevertheless, the *Infant Characteristics Questionnaire* (ICQ; Bates et al., 1979) has been widely used in numerous studies and demonstrates strong psychometric properties (Bates et al., 1979). Second, the variance due to shared methods can contribute to bias in the study, as the measures of key variables (maternal depression and anxiety symptoms and infant difficult temperament) are reported by the same individual, which should be considered in the interpretation of the results. However, the fact that these variables were assessed at different time points helps reduce the risk of shared method bias. Third, despite our large sample size, most of the participants identified as “White” and reported high levels of education and household income. Further work is thus needed in more diverse and representative samples. Fourth, we focused on the perceived partner support, which could differ from the support they receive. However, perceived support is the most studied aspect of social support and may be more linked to well‐being (Uchino, 2004; Yim et al., 2015). The literature examining the amount of support women receive during the perinatal period is limited, and existing evidence shows mixed outcomes (Yim et al., 2015). Importantly, while the study is longitudinal, its correlational nature prevents us from establishing causal links. Future studies using transactional models, such as cross‐lagged panel models, with repeated measures of mental health, partner support and infant temperament would also help better understand the directionality of the effects detected here. While we focused on two relevant mediators, we did not consider other potential pathways such as through parenting style, parental stress or the nature or quality of the parent‐child relationship. Finally, we were unable to include partner information beyond what was reported by the mother.

### 4.2 Implications for practice and/or further research

The perinatal period is a time of heightened vulnerability for mothers, making it crucial to closely monitor maternal mental health across pregnancy and into the postpartum period. Early identification of psychological distress, including symptoms of both depression and anxiety, can help direct mothers to appropriate resources and support systems when needed. Our findings reinforce the importance of integrating routine screening for maternal distress not only during pregnancy but also following childbirth, as postnatal symptoms emerged as a key mechanism linking prenatal distress to infant temperament outcomes. Moreover, our results highlight the potential protective role of perceived partner support. Perinatal interventions should therefore consider involving partners, promoting co‐parenting support and equitable caregiving practices, which may in turn foster maternal well‐being and optimal child development. Future studies may benefit from including multi‐informant or observational measures of infant temperament to complement maternal reports and reduce potential reporting biases. Longitudinal designs extending beyond the first year postpartum are also needed to examine the long‐term associations between prenatal and postnatal maternal mental health and various aspects of child development. Finally, investigating other plausible mediators such as parenting behaviors, parental stress, or mother–infant bonding would further clarify the mechanisms underlying these intergenerational links.

## 5 CONCLUSION

In conclusion, our findings revealed an association between maternal depression and anxiety symptoms during the third trimester of pregnancy and higher scores for infant difficult temperament at 12 months. Our study also showed that this association is indirect, mediated through two distinct pathways: via maternal depression and anxiety symptoms and lower perceived partner support at three months. These findings highlight the importance of considering not only prenatal symptoms but also postnatal symptoms as well as other variables such as perceived partner support when investigating the links between maternal symptoms and child development. This study contributes to the growing evidence on the intergenerational impact of maternal mental health, reinforcing the need for comprehensive perinatal mental health interventions.

## CONFLICT OF INTEREST STATEMENT

The authors declare no conflicts of interest.

## Data Availability

The data that support the findings of this study are available on request through a data access protocol from the corresponding author, C.M. Herba (herba.catherine@uqam.ca). The data are not publicly available due to privacy or ethical restrictions.
